# Why and When Temporary Workers Engage in More Counterproductive Work Behaviors with Permanent Employees in Chinese State-Own Enterprise: A Social Identity Perspective

**DOI:** 10.3390/ijerph19138030

**Published:** 2022-06-30

**Authors:** Xiaolang Liu, Chuanyan Qin, Shanshi Liu, Wenzhu Lu

**Affiliations:** 1 School of Management, Guangdong University of Technology, Guangzhou 510006, China; liuxiaolang0507@163.com; 2 School of Business Administration, South China University of Technology, Guangzhou 510006, China; bmssliu@scut.edu.cn (S.L.); 13073063700@163.com (W.L.); 3 School of Medical Business, Guangdong Pharmaceutical University, Guangzhou 510006, China

**Keywords:** employment status, organizational identification, counterproductive work behavior, turnover intention, temporary employee

## Abstract

Why do temporary workers sharing the same working conditions as permanent employees still frequently engage in deviant behaviors that negatively affect the organization’s interests? Drawing on the theory of social identity, this articlr discusses the relationships among employment status, organizational identification, and counterproductive work behavior. Time-lagged data were collected from sample of 210 dyads of employees and corresponding supervisors from a large Chinese state-owned service company, to test hypothesis. Results showed that temporary workers engage in counterproductive work behaviors more frequently than permanent employees, and organizational identification plays a mediating role in this process. Turnover intention moderated the relationship between employment status and counterproductive work behavior (organizational identification). In terms of turnover intention, organizational identification and counterproductive work behavior, two types of employees did not exhibit a significant difference. However, when turnover intention increase, there was a sharper decline in organizational identification and a greater increase in counterproductive work behaviors among temporary employees than among permanent employees. Finally, the theoretical and practical implications of these findings and future research directions are discussed.

## 1. Introduction

Hybrid employment has been a go-to choice for organizations to decrease costs and acquire needed talent urgently [1]. Research has reported that more than 80% of enterprises reorganize their workforces flexibly, and most companies employ more than 40% of nonstandard employees [2,3]. The same phenomenon has been found in China, and the proportion of temporary employees in China has continuously increased in recent years. The hybrid employment model, which includes temporary and permanent employees, has created many issues, as demonstrated by practice and research. In recent years, temporary employees in China have displayed several common destructive behaviors, such as changing customer data at random, using unsanitary treatment of ingredients. Despite the suspicion that these workers were declared temporary employees after they made mistakes, two unaddressed questions arise: Are temporary employees really demonstrating more sabotaging behaviors than permanent employees in the workplace? If so, why, and when does it happen? Thus, further verification is required to determine whether there exists a difference between the two types of employees’ engagement in counterproductive work behavior (CWB) and how these behaviors arise.

Previous research has suggested that nonstandard employment models might bring unfavorable outcomes to temporary employees and organizations due to the awful working conditions for nonstandard employees. For instance, contract employees or temporary workers have been associated with deviant behavior [4]. According to the theory of social exchange, researchers revealed that part-time employees showed lower initiative to launch OCBs than full-time employees in restaurants [5], both preferred work status and organizational culture play moderating roles in the process of bringing on adverse behaviors. From the perspective of the psychological contract, research on nonstandard employees demonstrated that they show a poorer job attitudes (e.g., lower job satisfaction and affective commitment) and lower psychological well-being [6]. However, relatively little is known about why atypical employees display certain behaviors and what factors can attenuate their negative effects. Accordingly, it is unclear what constitutes critical moderators and mediators of the employment status effect on behavioral outcomes. Recent reviews have suggested that the potential negative effects of hybrid employment on productivity-related outcomes are still poorly understood [1,7]. Especially with improvements in labor legal systems, incluing ‘equal pay for equal work’, temporary workers and permanent employees are increasingly enjoying the same work treatment in China. In this case, will contract workers engage in more CWB?

Reviewing prior research, we contend that there are several critical gaps in the hybrid employment literature that may contribute to this lack of clarity. First, the literature has largely ignored the diversification of current mixed employment situations. For instance, employees of different employment forms working in the same place or even the same job position, and enjoy the same working conditions instead of the poor working environments for nonstandard workers described in earlier literature [8]. Second, several theories, such as social exchange theory, conservation of resource theory and the psychological contract, were used to explain the behavioral differences between standard and nonstandard employees due to divergent exchange relationships between employees and the organization. Yet, when work conditions and other factors between standard and nonstandard employees are equalized, the assumption of nonstandard employment tends toward the idea that the economic exchange relationship no longer exists. Therefore, it is necessary to applied other perspective to explain the multi-employment relationship and the psychological and behavioral differences between employees in this new situation. In addition, the literature lacks an integrated conceptual framework that explains the unique psychological nature of nonstandard employment. Examinations of theory-based mediators and moderators that unpack when, how, and why nonstandard employees engage in certain work behaviors have been very rare [9,10]. Accordingly, theoretical integration is needed to further explicate when, how, and why nonstandard employees engage more in CWB.

This article sets out to investigate the relationship between employment status and CWB by virtue of social identity theory. We first focus on the mediating role of organizational identification, which is defined as “perception of oneness with or belongingness to an organization, where the individual defines him or herself in terms of the organization(s) in which he or she is a member” [11]. Drawing on social identity theory, when employees perceive that they are part of the organization, they establish their self-concept, subsequently allowing them to identify with the organization and finally improve their positive attitude and constructive behaviors [12]. Because of the employment status differences between temporary employees and permanent employees, they may show differences in organizational identification and CWB. We then examined the impact of turnover intention on the process of displaying CWB. From the perspective of social identity theory [13], organizational identification results in a construction process that is affected by many factors. In a multi-employment situation, temporary employees have a high planned turnover intention due to a fixed contract duration [14]. Those with uncertain jobs will not invest too many personal resources to establish deep employee–organization relationships. When employees conceive of an internal intention to leave, it generates an effect that influences their psychological state and behavior [15].

This article contributes to the literature in these aspects. First, while some research has explored the relation between employment status and behavior [16,17], it remains unclear what affects the divergence in CWB. From the perspective of social identity theory, we explored the important role of employment status in affecting employees’ CWBs by influencing their organizational identification. Second, although there exist studies that explored moderators and mediators between employment status and outcomes [18,19], little research has developed theoretically complex models that simultaneously examine the processes and boundary conditions of these linkages [20]. We extend this field by using social identity theory to explore when and why employment status affects CWB. In doing so, we analyze whether two kinds of employees diverge in terms of organizational identification and CWBs, and whether this divergence is conditional upon their turnover intention. Finally, we probe into employees’ perceptions of their affiliations in the scenario of a Chinese state-owned enterprise.

## 2. Theory and Hypothesis

### 2.1. Employment Status and Counterproductive Work Behavior

Employment status signals the category that an individual or organization occupies within a well-defined social hierarchy [21]. Researchers have proposed various classifications of employment status. In this article, we adopt the viewpoint that employment status includes permanent and temporary job statuses [22]. Individuals in higher positions, based on occupational prestige, represent social esteem in terms of positive privileges, while individuals in lower positions are affected by the opposite [23]. Research has shown that a wide range of phenomena within organizations could be explained by divergence in status [24]. For example, numerous studies have revealed that employment status is positively associated with contract fulfillment [25], job satisfaction [26], and commitment [27]. Researchers found that employment status was negatively related to employees’ working hours [28]. As such, assessing employment status seems to be a very plausible approach to understanding behaviors.

CWB refers to voluntary employee behaviors that are viewed by the organization as contrary to its legitimate interests, violate significant organizational norms, and threaten the well-being of the organization or its members [29]. In this article, we claim that employment status (permanent vs. temporary) can predict CWB. First, compensation, opportunities for training, and promotions for temporary employees are often inferior to those of permanent employees, which could elicit behaviors that are detrimental to the organization. Second, temporary employees are easily monitored and replaced, and often perform work that is peripheral to an organization’s main activities [30]. They always face higher levels of contextual constraints that prevent them from translating ability and effort into job performance in the context of performing tasks or engaging in adverse behaviors [31]. According to the stress theory, perceived injustice and situational constraints create stressful work conditions, which are positively related to CWB [32,33]. Past research has clearly shown that work stressors and negative emotional reactions are positively related to CWB [6,34].

In summary, temporary employees are likely to respond with a higher level of CWBs than permanent employees. This leads us to the following hypothesis:

**Hypothesis** **1:** *Temporary employees demonstrate more CWB than permanent employees*.

### 2.2. Employment Status and Organizational Identification: Social Identity Explanation

Several perspectives (e.g., psychological contracts and social exchange theory) have been employed to conceptualize employment status and its impact on organizationally and individually valued outcomes, such as workplace cohesion and work engagement [1,4,10]. Inconsistent with prior research, we argue that the social identity framework can capture the essence of diverse employment situations in the same work condition such that perceptions of employment type encapsulate an employee’s perceived status in an organization, wherein nonstandard employees are seen as inferior. No less importantly, this perspective also captures how well membership needs are satisfied.

Researchers have suggested that identity issues are salient, especially in the organizational setting, in the current contexts characterized by fragmentation, discontinuity, and economic crises [35]. In fact, the workplace has an important role in supporting self-concept in terms of social identification within an organization. People tend to classify themselves and others into various social groups, as described by social identity theory [36]. Organizational identification occurs when employees perceive oneness within an organization and feel that they belong to it. This process of incorporating the perception of oneself as a member of a specific organization into one’s general self-definition draws on the social identity perspective, which is the most pervasive theoretical framework used in contemporary organizational identification research [37].

Following social identity theory, we suggest that employment status communicates employees’ identity-relevant information related to their status and prospects within the organization because it defines whether they feel as though they are valued members of the organization. In fact, temporary employees with fixed terms usually perceive high job insecurity, which concerns the perception of an involuntary and undesired change in the continuity of the work situation [38]. Consequently, it seems reasonable to assume that these employees cannot feel that they are important members of the organization because they are worried about being excluded [39]. Specifically, this occurs as a reaction to the perceived threat to the needs for belonging, inclusion, and recognition [40]. Identification with an organization satisfies this range of human needs, which generate a sense of membership. Conversely, the frustration with these needs sparks concerns about how individuals think of themselves as members of their organization. Temporary employment with a fixed term does not fulfil membership needs because employees are worried about the future of their jobs or being a low-status member of the group [41]. Therefore, employment status represents a contextual factor that can affect the level of employees’ organizational identification from a social identity perspective.

### 2.3. Mediating Effects of Organizational Identification

According to social identity theory, social identification is ‘part of an individual’s self-concept which derives from his knowledge of his membership of a social group together with the value and emotional significance attached to that membership’ [12]. Organizational identification has been defined as a cognitive construct (awareness of membership), evaluative construct (sense of value), and affective construct (emotional investment) [42] that jointly describes the perceptions of oneness or belongingness to an organization [43]. Individuals who are satisfied with their fundamental needs, including self-enhancement, other self-related motives, and basic human needs, are likelier to identity with their organizations [42,44].

We argue that temporary employees have a lower level of organizational identification than permanent employees for several reasons. First, since temporary employees sign agreements with organizations to provide services for a specific length of time, making them feel powerless and unable to expect continuity and predictability in the workplace, this prevents employees from establishing identification with the organization [43]. Previous studies have also provided empirical support that emphasized that the limited-term contract results in a low level of organizational identification [45]. Second, individuals are motivated by the basic need to connect with others and to seek belonging within an organization [42]. However, temporary employees have less contact with the core persons in the organization [30], which results in socially isolated and alienated employees [46]. The process of externalization is based on limited-term employment, such as ‘taking the employees back out’ of their organizations through a weakening of the “attachments between employees and organizations” [47,48], which is an antecedent of organizational identification [45]. Third, most temporary employees work in the periphery of a company [14], which signals that they are perceived as disposable, replaceable, and interchangeable [49]. Furthermore, managers believe that temporary employees produce limited value [50]. Thus, temporary employees are inclined toward low self-effectiveness in an organization. The basic need for achievement is not satisfied by the organization, which makes it difficult for them to identify with the organization based on self-determination theory [51]. Hence, compared to permanent employees, temporary employees may identify less with the organization.

Furthermore, we argue that low levels of organizational identification could facilitate employees’ CWB. Organizational identification involves essential definition of self-concept, which can explain individual behavior in an organization [52]. At the core of the social identity approach to organizational behavior lies the notion that group membership is self-definitional to a greater or lesser degree [53]. People who identify with their organization tend to see themselves as personifying the organization. The more individuals identify with an organization, the more the organization’s values, norms, and interests are incorporated into the self-concept [42]. High identification encourages employees to devote more effort to benefiting the organization. On the contrary, individuals who have low levels of identification act based on the principle of egoism rather than organization. Their willingness to offer affective and behavioral support to the organization is lower. Prior researchers have found that low levels of organizational identification have negative effects on individual motivation [54], which is related to absenteeism and organizational citizenship behavior [55,56]. Thus, based on the above reasoning, we propose that organizational identification is negatively associated with CWB.

Putting together these derivations that organizational identification mediates the negative relationship between employment status and CWB, we propose the following:

**Hypothesis** **2:** *Organizational identification mediates the relationship between employment status and CWB*.

### 2.4. Moderating Role of Turnover Intention

Turnover intention is conceived to be a deliberate willfulness to leave the organization [57]. It has been described as the last withdrawal cognitions, a set to which thinking of quitting and intent to search for alternative employment. Turnover intention is related to organizational member relationship state, a powerful predictive index of turnover behavior [58,59]. When an employee wants to leave an organization, his or her relationship with the organization deteriorates quickly. Drawing from social identity theory, we propose that the existing divergence in turnover intention between the two types of employees exhibited by their psychological states and behavior continue to change.

Social identity theory posits that individuals will compare themselves to groups with the same characteristics, and then rank their social environment by establishing meaningful group categories. Finally, individuals will generate affiliation and identification with the group they belong to [11,53]. Similarity, employees possess different levels of affiliation to the organization depending on the subgroup they belong to. And when they have the intention to leave, they will gradually detach their identification from the organization that they will no longer regard themselves as part of the organization, and then change their attitudes and behaviors in the workplace [15].

Specifically, temporary employees are left in the periphery, typically receive less supervision and support from organizations, and are associated with less employee trust and lower commitment compared to permanent employees [30,60]. A strong tendency to leave would reinforce temporary employees’ recognition of their status, which would abate their identification with the organization and then increase their CWB. For permanent employees, however, they have experienced higher job satisfaction and stronger organizational commitment [26,27]. Even if have the intention to leave, these experience will act as a cushion that prevent them from a rapid decline in organizational identification. Accordingly, temporary workers who suffer more negative feelings than permanent employees would experience a sharper decrease in their organizational identification levels and demonstrate more CWB if they feel the desire to leave. Therefore, we propose the following:

**Hypothesis** **3a:** *Turnover intention moderates the relationship between employment status and organizational identification. When turnover intention is high, the difference in organizational identification between the two types of employees is greater, such that temporary employees’ organizational identification is even lower*.

**Hypothesis** **3b:** *Turnover intention moderates the relationship between employment status and CWB. When turnover intention is high, the difference in CWB between the two types of employees is greater, such that temporary employees’ CWB is even more pronounced*.

### 2.5. Moderated Mediating Effects

**Figure 1:**
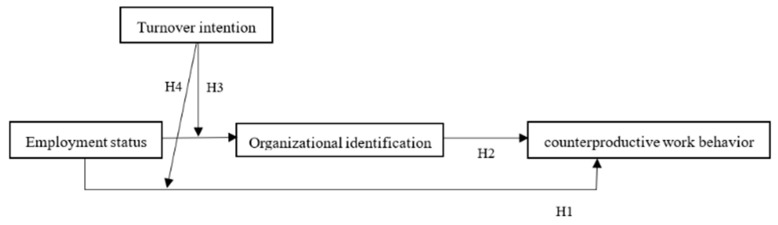
The hypothesized mediation and moderation model.

As previously mentioned, a stronger relationship between employment status and CWB will appear for high-level turnover intention employees. Furthermore, we propose that the indirect effect of employment status on CWB via organizational identification also be stronger for high-level leave intention employees. Specifically, when an employee has a high intention to leave, the indirect effect of employment status and CWB should be stronger. However, when an employee does not want to leave the organization, the indirect effect of employment status on CWB via organizational identification will be weaker. Figure 1 depicts the theoretical model. Consequently, we propose the following:

**Hypothesis** **4:** *Turnover intention moderates the mediating effect of organizational identification on the relationships between employment status and CWB, such that the indirect effect of employment status and CWB via organizational identification is stronger for high-level leave intention employees than for low-level leave intention employees*.

## 3. Methods

### 3.1. Sample and Procedure

Data were obtained from both permanent and temporary employees of a state-owned service company in China. Survey packets were sent separately to respondents and their direct supervisors. Attached to the survey instrument for both supervisors and their subordinates was a notice that explained the objective of the survey and ensured that their participation in the survey was voluntary. Furthermore, we explained to all respondents that their answers would be used only for the purposes of the survey. At time 1, employees were asked to report their post number (to determine their employment status from the human resources [HR] manager), organizational identification, turnover intention, POS, pay satisfaction, and demographic information. Their direct supervisors were asked to report subordinates’ CWB two weeks later.

With the assistance of the HR department, a member of the research team distributed two separate questionnaires to 280 employees and their 126 direct supervisors. We randomly selected 217 employees distributed among various departments, including management, service, marketing, and flight. After deleting some unmatched supervisor–subordinate dyads, we finally obtained 210 completed valid questionnaires.

210 employees and their 116 corresponding supervisory ratings of CWB matched surveys were retained for data analysis. Out of the 210 respondents, 108 (51.43%) were permanent employees with the remaining 102 employees on contract (48.57%). More than half of the participants were male (57.14%). 82.24% of the participants were younger than 35 years (47.14% were 25 years old or below, 38.10% were 26~35, 14.28% were 36 ~ 45, 0.48% were 46 years old and above), with an average job tenure of 6.18 (6.67% have tenure below 1 year, 23.81% were 2–3 years, 13.81% were 3–5 years, 17.62% were 5–7 years, 38.10% were more than 7 years). The respondents were fairly well- educated (7.4% of employees had middle school or lower education, 16.19% had high school and technical secondary school education, 76.19% had college or above education).

### 3.2. Measure

For all measures, respondents rated the items on a 5-point scale, ranging from 1 = strongly disagree to 5 = strongly agree. We translated English scales into Chinese, and then back-translated them, to ensure the equivalence, complete item information can be seen in Appendix A.

Employment status. Employment status was measured in a dichotomous variable, respondent was required to answer whether they belong to temporary employees (coded 0) or permanent employees (coded 1).

Organizational identification. Organizational identification was measured by six-items scale [11]. Sample items included ‘When someone praises organization, it feels like a personal compliment.’ and ‘If a story in the media criticized my organization, I would feel embarrassed’. The scale’s alpha reliability in this study is 0.909.

Counterproductive work behavior. A 12-item subscale was used to measure workers’ deviant behavior to organization [29]. Example items is ‘Taken property from work without permission’. The scale’s alpha reliability in this study is 0.956.

Turnover intention. The three-items scale was used to measure turnover intention [57]. Sample items included ‘I often think about quitting my job with my present organization’ and ‘I will probably look for a new job within the next year’. The scale’s alpha reliability in this study is 0.880.

Pay satisfaction. We use 18-items to measure employees’ pay satisfaction [61]. Sample items included ‘the raises I have typically received in the past’ and ‘amount the company pays toward my benefits’, Cronbach’s alpha for the scale was 0.893.

Perceived organizational support. And we use eight-items scale to measure employees’ perceived organizational support [62]. Sample items included ‘the organization appreciates any extra effort from me’ and ‘the organization takes pride in my accomplishments at work’. The scale’s alpha reliability in this study is 0.907.

Control variables. Following prior research, the demographic control variables measured were gender (1 = male, 2 = female), age (1 = under 25 years, 2 = 25–35 years, 3 = 35–45 years, 4 = 45–55 years, 5 = above 55 years), and income level (1 = under ¥2500, 2 = ¥2500–4500, 3 = ¥4500–6500, 4 = ¥6500–8500, 5 = above ¥8500), education (1 = middle school or below, 2 = high school or secondary school, 3 = junior college, 4 = bachelor degree), we also controlled the length of tenure (1 = within 1 year, 2 = 2–3 years, 3 = 3–5 years, 4 = 5–7 years, 5 = more than 7 years).

## 4. Results

### 4.1. Confirmatory Factor Analysis

We analyzed the data using MPLUS 7.0. Before testing our hypotheses, we used confirmatory factor analysis (CFA) to testify the discriminant validity of the key variables. We examined a baseline model that contains three factors; namely, organizational identification, turnover intention and CWB. CFA results of the test of our measurement model revealed support for this baseline model: χ^2^(df) = 2.310, *p* < 0.001, SRMR = 0.043, CFI = 0.946, TLI = 0.937, and RMSEA = 0.079. Compared to the 2-factor model (χ^2^(df) = 4.645, *p* < 0.001, SRMR = 0.082, CFI = 0.848, TLI = 0.825, RMSEA = 0.132) and 1-factor model (χ^2^(df) = 6.809, *p* < 0.001, SRMR = 0.094, CFI = 0.756, TLI = 0.721, RMSEA = 0.166), as seen in Table 1. In addition, the analysis results of convergent validity are displayed in Table 2, so the baseline model fits our data best.

### 4.2. Descriptive Statistics and Correlations

Table 3 presents the means, standard deviations of all key variables. Before combining the samples, we compared the temporary employees and permanent employees on means of the turnover intention, organizational identification, counterproductive work behavior. As we can see from Table 3, the organizational identification of permanent employees was higher than that of temporary employees, and the temporary employees’ turnover intention, counterproductive work behavior was higher than the permanent employees. Consistent with our predictions, temporary employees demonstrated less organizational identification (r = 0.218, *p* < 0.01), and more counterproductive work behavior (r = −0.302, *p* < 0.01); organizational identification is negatively correlated with counterproductive work behavior (r = −0.66, *p* < 0.01).

### 4.3. Hypothesis Testing

Hypothesis 1 predicted that temporary employees demonstrated more in counterproductive work behavior. As shown in Model 4 in Table 4, after controlling for demographic variable, employment status show a significant negative influence on counterproductive work behavior (β = −0.278, *p* < 0.01), indicates permanent employees show less counterproductive work behavior than temporary employees, which supporting hypothesis 1.

Hypothesis 2 proposed organizational identification as a mediator between employment status and counterproductive work behavior. As seen in model 2 and 4, we verify that employment status was associated with organizational identification (β = 0.233, *p* < 0.05) and counterproductive work behavior (β = −0.278, *p* < 0.01) respectively. After adding organization identification into model 4, it can be found that the negative relationship between employment status and counterproductive work behavior has declined (β = −0.171, *p* < 0.05), indicates that organization identification partly mediates the relationships between employment status and counterproductive work behavior. Besides, the results of soble test show that the indirect effect from employment status to counterproductive work behavior via organizational identification is significant (β = −0.131, S. E. = 0.043, 95% C. I. = [−0.202, −0.060]), further support Hypothesis 2.

**Figure 2:**
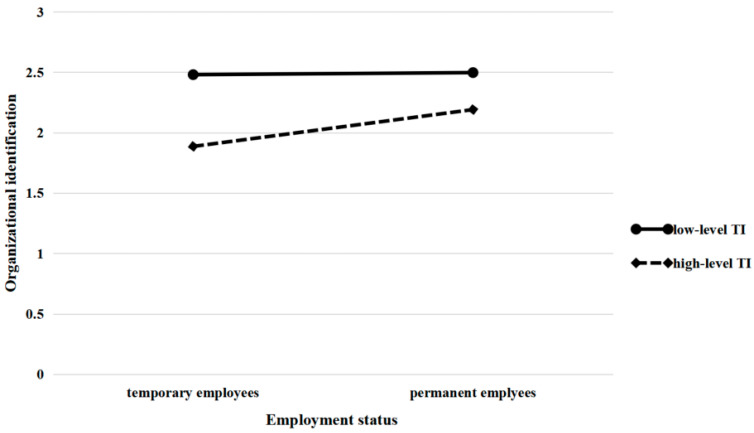
Interaction between employment status and turnover intention on organization identification.

Hypothesis 3a predicted that turnover intention moderates the direct effect of employment status on organization identification. As shown in model 2 of Table 5, the interaction term of employment status and turnover intention show a positive influence on organization identification (β = 0.155, *p* = 0.057 < 0.1). The interaction effect of employment status and turnover intention for organization identification is depicted in Figure 2. Besides, results showed that the positive relationship between employment status and organization identification was the greatest when turnover intention was in +1 SD level (β = 0.497, S. E. = 0.261, 95% C. I. = [0.067, 0.926]), weak when turnover intention in medium level (β = 0.353, S. E. = 0.185, 95% C. I. = [0.048, 0.658]), while the least when turnover intention was in −1 SD level (β = 0.209, S. E. = 0.110, 95% C. I. = [0.028, 0.390]). Hence, the hypothesis 3a was supported.

**Figure 3:**
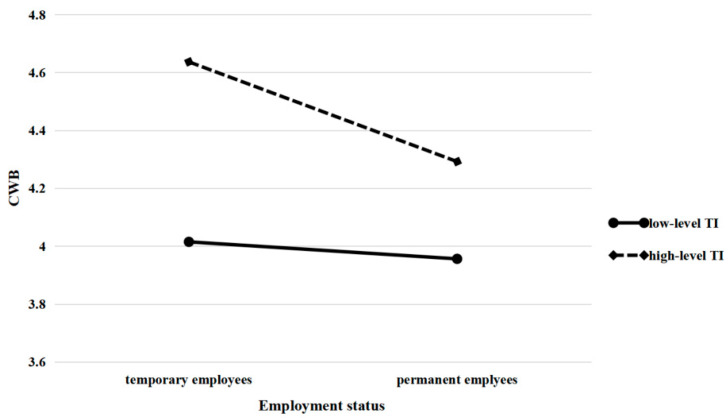
Interaction between employment status and turnover intention on CWB.

Hypothesis 3b predicted that turnover intention moderates the direct effect of employment status on counterproductive work behavior. It can be seen in model 4 of Table 5, the interaction term of employment status and turnover intention has a negative effect on counterproductive work behavior (β = −0.154, *p* < 0.05), the moderating effect can be seen in Figure 3. What’s more, results showed that the negative effect of employment status on counterproductive work behavior was the strongest when turnover intention was in +1 SD level (β = −0.493, S. E. = 0.215, 95% C. I. = [−0.847, -0.139]), weak when turnover intention in medium level (β = −0.350, S. E. = 0.153, 95% C. I. = [−0.602, −0.099]), while the weakest when turnover intention was in −1 SD level (β = −0.208, S. E. = 0.091, 95% C. I. = [−0.357, −0.059]). Hence, the hypothesis 3b was also supported.

Hypotheses 4 supposed that the mediation of organizational identification on counterproductive work behavior varies as a function of turnover intention. As shown in Table 6, the confidence interval of index for the moderated mediation was significant (β = −0.099, S. E. = 0.042, 95% C. I. = [−0.168, −0.030]), indicates that the indirect effect from employment status to counterproductive work behavior was moderated by turnover intention. Besides, the conditional indirect effects were insignificant when the level of turnover intention is 1 standard deviation (SD) below the mean value (β = 0.016, S. E. = 0.054, 95% C. I. = [−0.072, 0.104]). However, the conditional indirect effects were significant when the level of turnover intention is 1 standard deviation (SD) above the mean value (β = −0.168, S. E. = 0.056, 95% C. I. = [−0.260, −0.077]), or at mean value (β = −0.076, S. E. = 0.038, 95% C. I. = [−0.139, −0.013]). The result illustrated that when perceiving medium or high turnover intention, temporary and permanent employees will demonstrate a significantly difference in their organizational identification and counterproductive work behavior. Thus, hypothesis 4 was supported.

## 5. Discussion

The purpose of this article was to investigate when and why different employment status employees’ (temporary workers vs. permanent employees) behaviors differ in terms of CWB in the Chinese state-owned enterprise context. We first investigated whether and why there exist differences in CWBs amid different employment statuses. The results of the study confirmed our hypothesis that employment status has an impact on employee CWB through organizational identification. Temporary workers’ organizational identification was found to be lower than that of permanent employees, and their CWB was higher. Previous research focused on two employment statuses in the workplace to determine deviations in behavior, mostly from the economic and social exchange perspectives [4,19]. For most atypical employees in marginal positions, their working conditions are poor. Their relationship with the organization is transactional, easily causing them to feel unfairly treated, which triggers negative behaviors. For fixed-term contract workers, their career development is very limited. They are difficult to integrate into the company’s core group to build deep employee–organization relationships. When employees are on the edge of the organization, it is difficult to form self-concepts. Thus, they tend to engage in behaviors that deviate from the organization’s values as well as behaviors that betray the interests of the organization.

The results indicate that organizational identification plays a mediating role in the relationship between employment status and CWB. Various employment models result in differences in employees’ identification with the organization, which could exert an influence on their CWBs. The results imply that the reason for temporary employees’ frequent destruction of the workplace is a lack of organizational identification. Employment status communicates to employees’ identity-relevant information related to their status and prospects within the organization because it defines whether they feel valued as members of the organization. Temporary workers are the “second-order members” of an organization compared to permanent workers. Consequently, it seems reasonable to assume that these employees do not feel valued by the organization [38], which inhibits identity formulation. In addition, for temporary employees, job instability and feelings of powerlessness lead to low organizational identification. As such, they may be less hesitant to display more CWB. Existing research has investigated the critical role of employment status on employees’ behaviors mainly through social exchange theory and psychological contract theory [5,63], and we expanded the understanding of employment status’s impact on behaviors by drawing on social identity theory.

Another aim of this article was to explore the boundary conditions in the process by which employment status takes effect in manifesting CWB. The results demonstrated that there is an interaction effect between employment status and turnover intention on CWB. As can be seen in Figure 2, temporary employees who report intense turnover intentions experience a greater increase in CWBs. Temporary employees with higher turnover intentions who participate in more CWB may do so for the following reasons: first, as the psychological connection between these contract employees and the organization is weak (as can be seen from their high turnover intention), they tend to violate the interests of the organization; second, due to their fixed-term contracts, the probability of actual departures is relatively high, the organization’s constraints of norms have limited influence on them. Once they truly leave, dispensing with responsibilities will also stimulate employees’ motivation to engage in CWB.

Furthermore, turnover intention moderates the relationship between employment status and organizational identification, which in turn affects employees’ CWBs. Figure 3 shows that temporary employees who have a strong intention to leave experience a bigger decline in organizational identification than permanent employees, which could affect their CWBs. In contrast, permanent employees’ organizational identification and CWB show a gentler change when their turnover intention varies. The reason is that, except for formal status, permanent employees generally experience higher job satisfaction and positive psychological contracts and are treated better than temporary employees in terms of compensation and benefits [5,64]. Although they do not plan to be members of the organization in the future, organizational identification built on positive psychological factors will be maintained for the necessary period. For temporary employees, however, inferior treatment may result in their feeling ostracized. Once they decide to resign, their low-level organizational identification deteriorates quickly [32], which subsequently elicits CWBs.

The analysis results of the moderated mediating effect suggested that when they do not intend to leave, the level of organizational identification and CWB do not differ vastly between temporary employees and permanent employees (β_oi_ = 0.016, 95% C. I. = [−0.072, 0.104]), as shown in Table 6, which contrasts earlier literature [42,65]. The following three reasons also provide an explanation: (1) Unlike some on-demand employees who work at home, temporary employees in this study usually worked together with permanent employees, so both employees may foster organizational identification to a similar degree; (2) temporary employees who have no intention to leave are always inclined to change their status and transform into regular employees in the organization. This expectation may increase their organizational identification, which is originally slightly lower than that of permanent employees; and (3) Temporary employees may restrain themselves from engaging in deviant behaviors deliberately to obtain a formal position in the current organization. Moreover, it comes back to the previous result that there are differences in organizational identification and CWB between the two employment statuses. The reason may be that temporary employees have a relatively higher turnover intention than permanent employees (which can be seen in Table 3; the mean value of turnover intention is 2.484 for temporary employees and 2.077 for permanent employees) in our sample. The results confirmed that temporary employees are inclined to have a high level of turnover intention compared to permanent employees.

### 5.1. Theoretical Implications

Several theoretical implications can be noted. First, previous research revealed the impact of employment status’s impact on employees’ behavior, but mainly from the theoretical perspective of the psychological contract or social exchange theory [5,63,64]. From the angle of social identity, we investigated the emergence of CWB in hybrid employment and verified the completely mediating effect of organizational identification between employment status and CWB. As such, this study extends our understanding of why temporary and permanent employees show different levels of CWB in the workplace.

Second, we uncovered the mechanism by which employment status influences employees’ CWBs. Combining the perspective of employee–organization relationships and social identity, we explored the factors that affect employees’ CWB in hybrid employment. Analysis results showed that organizational identification acts as a cushion in manifesting CWB, especially for temporary employees, which provides empirical evidence that organizational identification is also needed for temporary employees to improve their behavior in the workplace [1,66].

Third, we revealed that turnover intention could act as a boundary condition, affecting the relationship between employment status and employees’ organizational identification and CWB. Most research on turnover intention regarding it as an employee behavior outcome has explored factors that could affect it [67,68,69,70]. This article argues that when employees are in the leave intention state, their psychology and behavior change. The results showed that turnover intention could influence employees’ organizational identification and CWB, which adds to the research on turnover intention. Furthermore, a former study proved that the planned turnover of temporary workers has an inverted U-shaped relationship with unit performance [14], increasing the costs of operational disruption [71,72]. This article provides evidence that the leave intention of temporary workers increases costs for organizations by increasing CWB.

Finally, we provide significant insight into diverse employment models in the Chinese state-owned enterprise scenario. Compared to temporary employees in private enterprises, this group has a longer average tenure and better working conditions, and they even get paid more. Therefore, when they do not have the intention to leave, they can form organizational identification at the same level as permanent employees. As such, exploring specific hybrid employment scenarios is important; otherwise, some key distinctions between temporary and permanent employees may be overlooked.

### 5.2. Practical Implications

This article proposes three suggestions for management practices. First, employment status has an influence on CWB [5,63,64]. According to the research results, temporary employees still have a greater tendency toward CWB than regular employees. Managers need to pay attention to the psychological behavior of temporary employees to avoid CWBs that bring irreparable losses to the organization.

Second, employment status influences CWB through organizational identification after controlling for other key psychological variables (POS and pay satisfaction). Temporary workers’ commitment or the identification with organization has been overlooked in the past [1,72,73]. If they do not establish proper organizational identification, they may fall into identity anxiety, causing the depletion of cognitive and emotional resources that then leads to counterproductive behaviors. Therefore, in addition to providing proper salaries and a fair working environment, it is important to help temporary employees establish organizational identification. Although it is very difficult to build organizational identification among temporary employees in a short and fixed working time, organizations and managers should make appropriate tradeoffs. Organizations can select nonstandard employees who demonstrate consistency with the organizational core values, provide opportunities for external employees to contact and communicate with the core employees, and help them construct their organizational identity. Our research has implications for the management of other atypical employees, such as independent professionals, agent employees, and freelancers.

Third, turnover intention could accelerate the decline of employees’ organizational identification as well as the increase in CWBs, especially for temporary employees with high levels of turnover intention. Managers must note that, due to fixed-term contracts, temporary employees are in a state of high-level turnover intention. On the one hand, organizations have to trade off gains and costs by using temporary employees. On the other hand, the opportunity for career development in an organization for temporary employees may protect some of them from “planned turnover,” which can slow down temporary employees’ turnover intentions and reduce CWB.

### 5.3. Limitations and Suggestions for Future Research

There are some limitations to this article. First, this article only examined employees’ CWBs against organizations under the influence of factors such as organizational identification and turnover intention. It did not consider CWB against individuals. Future research can remedy this limitation by considering both CWBs against organizations and individuals to obtain a comprehensive understanding of the impacts of employment status on employees’ CWB. Second, with a limited sample, it is difficult to probe, except for turnover intention, other external factors that can cause differences between the two types of employees. Third, this study was conducted in a Chinese state-owned company; future research needs to continue exploring hybrid employment in other special scenarios.

## 6. Conclusions

This article explored the relationship between employment status and CWB in the workplace as well as the role of organizational identification and turnover intention in this relationship in the framework of social identity theory. The results showed that the CWBs of temporary employees were significantly more frequent than those of permanent employees, and organizational identification played a mediating role. In addition, turnover intention and employment status have an interaction effect on employees’ organizational identification and CWB. When conceiving of a high turnover intention, the effect of employment status on organizational identification is greater and subsequently affects their CWB. Moreover, the direct effect of employment status on CWB would be stronger when employees have a high turnover intention.

## Figures and Tables

**Table 1:** Confirmatory factor analysis results.

Model	χ^2^(df)	SRMR	CFI	TLI	RMSEA
3-factor model	2.310 ***	0.043	0.946	0.937	0.079
2-factor model ^a^	4.645 ***	0.082	0.848	0.825	0.132
1-factor model	6.809 ***	0.094	0.756	0.721	0.166

Note(s): ^a^ turnover intention and organizational identification were combined into one factor; *** *p* < 0.001.

**Table 2:** Convergent validity analysis results.

Variables	AVE	C. R.	Reliability
Turnover intention	0.726	0.888	0.880
Organizational identification	0.609	0.886	0.909
Counterproductive work behavior	0.564	0.939	0.956

Note(s): AVE, Average Variance Extracted; C. R., convergent validity.

**Table 3:** Means, Standard Deviations, Correlations of variables.

	Temporary Employees		Permanent Employees											
	M	SD	M	SD	1	2	3	4	5	6	7	8	9	10
Gen	1.530	0.502	1.610	0.490										
Age	1.400	0.585	1.940	0.759	−0.155 *									
Inc	1.320	0.798	2.370	0.849	0.114	0.340 **								
Edu	2.750	0.938	3.190	0.751	0.330 **	−0.012	0.232 **							
Ten	2.750	1.156	4.330	1.102	0.077	0.523 **	0.315 **	0.135						
PS	3.142	0.682	3.220	0.585	−0.025	−0.037	0.100	0.051	−0.037					
POS	3.477	0.638	3.492	0.760	0.018	−0.003	0.016	−0.100	0.042	0.084				
ES	0.000	0.000	1.000	0.000	0.083	0.372 **	0.537 **	0.247 **	0.575 **	0.062	0.011			
TI	2.484	0.896	2.077	0.916	−0.017	−0.124	−0.201 **	−0.043	−0.135	−0.173 *	0.013	−0.220 **		
OI	3.529	0.699	3.815	0.582	0.080	0.091	0.199 **	0.063	0.071	0.436 **	0.067	0.218 **	−0.432 **	
CWB	2.638	0.643	2.264	0.541	−0.163 *	−0.096	−0.235 **	−0.162 *	−0.152 *	−0.518 **	−0.049	−0.302 **	0.499 **	−0.660 **

Note. N (Temporary employees) =102; N (Permanent employees) = 108. Gender coded: 1 = male, 0 = female. Inc, income level; Edu, education level; Ten, tenure; PS, pay satisfaction; POS, perceived organizational support; ES: employment status; TI, turnover intention, OI, organizational identification; CWB, counterproductive work behavior; * *p* < 0.05; ** *p* < 0.01.

**Table 4:** Mediating effect of organizational identification.

	OI		CWB		
	Model 1	Model 2	Model 3	Model 4	Model 5
Gender	0.124	0.130	−0.185 *	−0.192 *	−0.133 *
	(0.088)	(0.087)	(0.076)	(0.075)	(0.064)
Age	0.072	0.069	−0.046	−0.044	−0.012
	(0.068)	(0.067)	(0.059)	(0.058)	(0.049)
Income	0.081	0.037	−0.068	−0.016	0.001
	(0.046)	(0.050)	(0.040)	(0.043)	(0.036)
Education	−0.010	−0.025	−0.037	−0.019	−0.031
	(0.050)	(0.050)	(0.044)	(0.043)	(0.037)
Tenure	0.000	−0.037	−0.040	0.004	−0.013
	(0.035)	(0.039)	(0.031)	(0.034)	(0.029)
PS	0.442 ***	0.436 ***	−0.502 ***	−0.495 ***	−0.294 ***
	(0.064)	0.063	(0.056)	(0.055)	(0.051)
POS	0.024	0.025	−0.003	−0.004	0.008
	(0.057)	(0.057)	(0.050)	(0.049)	(0.042)
ES		0.233 *		−0.278 **	−0.171 *
		(0.109)		(0.094)	(0.081)
OI					−0.459 ***
					(0.050)

Note. N = 210. PS, pay satisfaction; POS, perceived organizational support; ES: employment status; OI, organizational identification; CWB, counterproductive work behavior. * *p* < 0.05; ** *p* < 0.01; *** *p* < 0.001 (two tailed).

**Table 5:** Moderating effects of employment status.

	OI		CWB	
	Model 1	Model 2	Model 3	Model 4
Gen	0.123	0.120	−0.185 **	−0.182 **
	(0.080)	(0.080)	(0.067)	(0.066)
Age	0.057	0.055	−0.031	−0.029
	(0.063)	(0.062)	(0.052)	(0.051)
Income	0.015	0.010	0.008	0.013
	(0.046)	(0.046)	(0.038)	(0.035)
Education	−0.017	−0.015	−0.028	−0.030
	(0.046)	(0.046)	(0.038)	(0.038)
Tenure	−0.039	−0.032	0.006	−0.002
	(0.036)	(0.036)	(0.030)	(0.030)
PS	0.380 ***	0.371 ***	−0.435 ***	−0.426 ***
	(0.059)	(0.059)	(0.049)	(0.049)
POS	0.036	0.030	−0.015	−0.009
	(0.053)	(0.052)	(0.044)	(0.043)
ES	0.170 *	−0.192	−0.211 *	0.149
	(0.102)	(0.216)	(0.084)	(0.178)
TI	−0.239 ***	−0.322 ***	0.255 ***	0.337 ***
	(0.041)	(0.060)	(0.034)	(0.049)
ES×TI		0.155 (*p* = 0.057 < 0.1)		−0.154 *
		(0.082)		(0.067)

N = 210. PS, pay satisfaction; POS, perceived organizational support; ES, employment status; OI, organizational identification; CWB, counterproductive work behavior. * *p* < 0.05; ** *p* < 0.01; *** *p* < 0.001 (two tailed).

**Table 6:** Result of moderated mediating effect.

TL	Employment Status→Organizational Identification→Counterproductive Work Behavior	
	Conditional Indirect Effects	Moderated Mediating Effect
−1 SD	0.016 [−0.072, 0.104]	−0.099 [−0.168, −0.030]
Mean value	−0.076 [−0.139, −0.013]	
+1 SD	−0.168 [−0.260, −0.077]	

Note. N = 210. TL, turnover intention, Results are based on 5000 bootstrap samples. Low limited confidence interval and upper limited confidence interval are in the brackets.

## Data Availability

The datasets used and analyzed in the current study are available from the corresponding author upon reasonable request.

## Appendix A

Organizational identification

[1 = Strongly agree; 5 = Strongly disagree]

1. When someone criticizes this company, it feels like a personal insult to me.
2. I am very interested in what others think about this company.
3. When I talk about this company, I usually say ‘we’ rather than ‘they’.
4. This company’s successes are my successes.
5. When someone praises this company, it feels like a personal compliment to me.
6. If a story in the media criticized this company, I would feel embarrassed.

(Mael, F.; Ashforth, B. E. Alumni and their alma mater: a partial test of the reformulated model of organizational identification. J Organ Behav. 1992, 13, 103–123. [11])

Counterproductive work behavior

[1 = Strongly agree; 5 = Strongly disagree]

1. This employee taken property from work without permission.
2. This employee spent too much time fantasizing or daydreaming instead of working.
3. This employee falsified a receipt to get reimbursed for more money than you spent on business expenses.
4. This employee taken an additional or longer break than is acceptable at your workplace.
5. This employee come in late to work without permission.
6. This employee littered your work environment.
7. This employee neglected to follow your boss’s instructions.
8. This employee intentionally worked slower than he/she could have worked.
9. This employee discussed confidential company information with an unauthorized person.
10. This employee used an illegal drug or consumed alcohol on the job.
11. This employee put little effort into your work.
12. This employee dragged out work in order to get overtime.

(Bennett, R. J.; Robinson, S. L. Development of a measure of workplace deviance. J Appl Psychol. 2000, 85, 349–360.) [29]

Turnover intention

[1 = Strongly agree; 5 = Strongly disagree]

1. I often think about quitting my job with my present organization
2. I will probably look for a new job within the next year
3. How likely is it that l will actively look for a new job in the next year?

(Aryee, S.; Chen, B. Z. X. Trust as a mediator of the relationship between organizational justice and work outcomes: test of a social exchange model. J Organ Behav. 2002, 23, 267–285. [57])

Pay satisfaction

[1 = Strongly agree; 5 = Strongly disagree]

1. I feel satisfied with my take-home pay.
2. I feel satisfied with my benefit package.
3. I feel satisfied with my most recent raise
4. I feel satisfied with influence my supervisor has on my pay.
5. I feel satisfied with my current salary.
6. I feel satisfied with amount the company pays toward my benefits.
7. I feel satisfied with the raises I have typically received in the past.
8. I feel satisfied with the company’ s pay structure.
9. I feel satisfied with information the company gives about pay issues of concern to me.
10. I feel satisfied with my overall level of pay.
11. I feel satisfied with the value of my benefits.
12. I feel satisfied with pay of other jobs in the company.
13. I feel consistency of the company’s pay policies.
14. I feel satisfied with size of my current salary.
15. I feel satisfied with the number of benefits I receive.
16. I feel my raises are determined.
17. I feel differences in pay among jobs in the company.
18. I feel satisfied with how the company administers pay.

(Heneman, III. HG.; Schwab, D. P. Pay satisfaction: Its multidimensional nature and measurement. Int J Psychol. 1985, 20, 129–141. [61])

Perceived organizational support

[1 = Strongly agree; 5 = Strongly disagree]

1. My organization cares about my opinions.
2. My organization cares about my well-being.
3. My organization appreciates any extra effort from me.
4. My organization would ignore any complaint from me.
5. Even if I did the best job possible, my organization would fail to notice.
6. My organization cares about my general satisfaction at work.
7. My organization shows very little concern for me.
8. My organization takes pride in my accomplishments at work.

(Shen, J.; Benson, J. When CSR is a social norm: How socially responsible human resource management affects employee work behavior. J Manage, 2016, 42, 1723–1746.)
